# In Vitro Osteogenic Stimulation of Human Adipose-Derived MSCs on Biofunctional 3D-Printed Scaffolds

**DOI:** 10.3390/biomedicines13112755

**Published:** 2025-11-11

**Authors:** Serena Munaò, Ugo D’Amora, Luana Vittoria Bauso, Alfredo Ronca, Paola Manini, Alessandro Pezzella, Maria Grazia Raucci, Luigi Ambrosio, Giovanna Calabrese

**Affiliations:** 1 Department of Chemical, Biological, Pharmaceutical and Environmental Sciences (ChiBioFarAm), University of Messina, Viale F. Stagno d’Alcontres 31, 98166 Messina, Italy; serena.munao@studenti.unime.it (S.M.); luanavittoria.bauso@unime.it (L.V.B.); 2 Institute of Polymers, Composites and Biomaterials, National Research Council, (IPCB-CNR), 80125 Naples, Italy; ugo.damora@cnr.it (U.D.); alfredo.ronca@cnr.it (A.R.); mariagrazia.raucci@cnr.it (M.G.R.); 3 Institute of Polymers, Composites and Biomaterials, National Research Council, (IPCB-CNR), 23900 Lecco, Italy; 4 Department of Chemical Sciences, University of Naples Federico II, 80126 Naples, Italy; pmanini@unina.it; 5 Bioelectronics Task Force, University of Naples Federico II, 80126 Naples, Italy; alessandro.pezzella@unina.it; 6 Department of Physics “E. Pancini”, University of Naples Federico II, 80126 Naples, Italy

**Keywords:** mesenchymal stem cells, bone regeneration, bioactive factors, natural polymer-based hydrogels, osteoconductive and osteoinductive properties

## Abstract

**Background:** Human adipose-derived mesenchymal stem cells (hADMSCs) are widely used in regenerative medicine due to their ability to proliferate and differentiate. Bone tissue engineering represents an innovative alternative to traditional grafts by combining biomimetic materials, stem cells, and bioactive factors to promote bone regeneration. Gellan gum (GG) is a promising scaffold material owing to its excellent biocompatibility and favorable physicochemical characteristics; however, chemical modifications such as methacrylation are necessary to enhance its mechanical strength and long-term stability. In this in vitro study, osteoprogenitor cells are cultured for 21 days on three 3D-printed GGMA-based scaffolds to evaluate their biological response: (i) neat GGMA, (ii) GGMA functionalized with hydroxyapatite (HAp), and (iii) GGMA functionalized with eumelanin derived from black soldier fly (BSF-Eumelanin). **Methods:** Cell adhesion, viability, proliferation and osteogenic differentiation are evaluated using MTT assays, histological staining (H&E and Alizarin Red S), alkaline phosphatase (ALP) activity, and gene expression analysis of key osteogenic markers. **Results:** Our results show that all GGMA-based scaffolds support cell adhesion, growth, and proliferation, while BSF-Eumelanin and HAp notably enhance osteogenic differentiation compared to neat GGMA. **Conclusions:** These findings highlight the potential of embedding bioactive factors into GGMA scaffolds to improve osteoconductive and osteoinductive performance, offering a promising strategy for bone repair.

## 1. Introduction

Mesenchymal stem cells (MSCs) are adult, nonhematopoietic, multipotent progenitor cells capable of self-renewal and differentiation into many cell types, including chondrocytes, adipocytes, tenocytes and cardiomyocytes and osteogenic lineage [1]. While MSCs can be isolated from numerous tissues, such as bone marrow, dental pulp, and peripheral blood, adipose-derived mesenchymal stem cells (hADMSCs) are a particularly valuable source for tissue engineering due to their accessibility and high yield [2,3]. The minimally invasive harvesting procedure via liposuction makes hADMSCs both practical and clinically relevant for applications like bone tissue engineering (BTE) [2]. Furthermore, hADMSCs demonstrate a robust capacity for proliferation and differentiation, guided by microenvironmental signals such as the extracellular matrix (ECM), growth factors, and mechanical signals [4]. In recent decades, BTE has emerged as an innovative approach within regenerative medicine. It aims to overcome the limitations of conventional bone grafts, such as donor site morbidity, limited availability, and potential immune rejection, by culturing stem cells on biocompatible biomaterials to guide bone regeneration [5].

Biomaterials play an active role in inducing specific cellular differentiation by providing a controlled biomechanical environment and supporting essential processes like nutrient and gas exchange. For BTE scaffolds, this requires materials with high biocompatibility, controlled degradability, suitable porosity and interconnectivity and excellent surface bioactivity [6]. In this scenario, three-dimensional (3D) (bio)printing has revolutionized BTE by enabling the precise fabrication of biomimetic scaffolds that replicate the complex architecture and hierarchical organization of native bone [7]. This additive manufacturing technique allows for the creation of customized, patient-specific implants and the integration of bioactive molecules, growth factors, or cells directly into the scaffold, thereby accelerating bone regeneration [8,9,10]. The structural, mechanical, and biological tunability offered by 3D printing helps bridge the gap between synthetic constructs and living bone tissue, advancing personalized regenerative therapies [9]. Various biomaterials, including bioceramics [11], metals [12,13], carbon-based nanomaterials [14], and both natural and synthetic polymers [15,16,17], have been investigated for BTE. However, conventional biomaterials often lack the necessary bioactivity for successful osteointegration, which can lead to regeneration failure [18]. Polysaccharide-based hydrogels, derived from natural polymers, have emerged as a promising alternative for BTE. Their excellent biocompatibility, high biodegradability, nontoxic degradation products, and flexible, hydrophilic nature allow them to mimic native ECM and support crucial cellular processes like adhesion, growth and differentiation [19,20]. Despite these advantages, natural polymers are often limited by poor mechanical properties, an uncontrollable degradation rate and low stability in physiological conditions.

Several polysaccharides, such as gellan gum (GG), alginate, and chitosan, are frequently used in tissue engineering due to their intrinsic bioactivity, biodegradability, and ability to mimic the ECM. These polymers can be chemically modified to enhance specific functionalities, including stability, swelling behavior, and mechanical properties [21]. Among them, GG, a linear, negatively charged exopolysaccharide derived from *Sphingomonas bacteria*, has gained considerable interest for BTE due to its favorable physicochemical properties and high biocompatibility [22]. Despite this, the reduced mechanical strength, poor long-term stability, and challenging gelation properties limit its use in 3D-printed scaffolds. To address these limitations, GG is commonly chemically modified via methacrylation, a process that improves its structural integrity and makes it a suitable bio-ink for 3D printing [23]. Methacrylated GG (GGMA) has already showed interesting properties for tissue engineering applications, as shown in our previous work [20]. A complementary strategy involves incorporating bioactive agents like hydroxyapatite (HAp), or biological molecules into GG-based scaffolds to enhance mechanical strength and improve osteoconductive and osteoinductive properties both in vitro and in vivo [24]. More recently, research has explored the use of eumelanin pigments extracted from the cuticles of black soldier flies (*Hermetia illucens*, BSF), BSF-Eumel, as a natural bioactive agent to improve the bone repair properties of 3D-printed scaffolds [20,25]. Eumelanin’s unique physicochemical properties, including strong redox activity, metal-binding capacity, and antioxidant effects, make it a promising component for BTE [26]. The catechol and indole groups within eumelanin can chelate calcium (Ca^2+^) and other ions, thereby promoting mineral deposition and osteogenic differentiation. Its antioxidant capacity protects cells from oxidative stress and stabilizes key osteogenic signaling pathways [27]. Furthermore, eumelanin’s negatively charged surface enhances protein adsorption and integrin-mediated cell adhesion. These combined physicochemical and biological features allow eumelanin to actively interact with cells, favoring adhesion, migration, proliferation and differentiation of stem cells [28,29].

Herein, we evaluate the biological response of hADMSCs cultured on 3D-printed GGMA scaffolds over 21 days. We compare the biocompatibility and osteoinductivity of GGMA scaffolds functionalized with BSF-Eumel and HAp against neat GGMA. Cell viability, adhesion, proliferation, and osteogenic differentiation were analyzed at several timepoints.

## 2. Materials and Methods

### 2.1. GGMA Synthesis and 3D Printing

GGMA scaffolds used in this study were developed as already previously described [14]. Briefly, low acyl GG (Gelzan, Sigma Aldrich, Milan, Italy) was functionalized by methacrylic anhydride (MA, purity ≥ 94%, Sigma Aldrich, Milan, Italy). The reaction was carried out for 6 h at 50 °C and 8 < pH < 8.4. A 20 mol % MA excess/unit of GG was used. Afterwards, GGMA was precipitated in cold ethanol (reagent grade, 98%, Sigma Aldrich, Milan, Italy), and purified by dialysis for 72 h to remove unreacted MA and ethanol residues. Three different inks were produced. (A) Neat GGMA ink was prepared by dissolving GGMA in water solution at a concentration of 4% *w*/*v*. 2-hydroxy-4′-(2-hydroxyethoxy)-2 methylpropiophenone (Irgacure 2959, gas chromatography area ≥ 98%, Sigma Aldrich, Milan, Italy) at a concentration of 5% *w_Irgacure_*/*w_GGMA_* was added, as crosslinking agent. (B) GGMA ink functionalized with BSF-Eumel was achieved by dissolving GGMA at the same concentration in a lower volume, containing Irgacure. The corresponding volume of BSF-Eumel, diluted in distilled water, was then added straight to the polymer solution to achieve the final concentration of 0.3 mg/mL. BSF-Eumel was produced by Insectta Pte. Ltd., a company located at 77 Ayer Rajah Crescent, #02-21/26, Singapore 139954, in accordance with UEN: 201809941M International Publication No.: WO/2021/183058. The BSF-Eumel was completely characterized in recent works [20,25]. (C) GGMA/HAp nanocomposite ink was produced by physical blending of GGMA and HAp. To this aim, HAp was synthesized as previously reported in our previous work [20]. Afterwards, GGMA was dissolved as previously described, and 30% *w_HAp_*/*w_GGMA_* HAp was added. The developed scaffolds were named as follows: (i) GGMA4, non-functionalized, used as control group; (ii) GGMA4/BSF-Eumel, functionalized with eumelanin and (iii) GGMA4/HAp30, blended with HAp. Table 1 summarizes the chemical composition of the inks.

The 3D printing was performed through “Rokit Invivo 4D2” (Rokit Healthcare Inc., Seoul, Republic of Korea). A needle with 0.41 mm inner diameter, 0.3 mm layer thickness, 45% infill density and a grid pattern were used to manufacture cylindrical samples (diameter (⌀) = 13 mm and thickness (t) = 3 mm). After printing, scaffolds were double crosslinked by exposure to UV light (Analytik Jena UVP crosslinker, Jena, Germany, λ: 365 nm, P: 10 J/cm^2^) for 10 min, and by dipping the scaffolds for 10 min, in 0.05% (*w*/*v*) CaCl_2_ at room temperature. Table 2 and Table 3 summarize the different conditions for GGMA-based inks processing and post processing.

The antioxidant ability of the scaffolds was assessed using the 2,2-diphenyl-1-picrylhydrazyl (DPPH) radical scavenging assay (Sigma Aldrich, Milan, Italy). A DPPH stock solution was prepared at a concentration of 39.5 mg/L in a 1:1 (*v*/*v*) ethanol-water mixture. For each test, 3 mL of this solution were added to the scaffold samples and incubated in the dark for 1.5 h to prevent light-induced degradation. The absorbance of each mixture was then measured at 517 nm using a UV–Vis spectrophotometer (SPECORD 210 PLUS Double-Beam, Analytik Jena, Jena, Germany). The DPPH radical scavenging activity (SA_DPPH_) was calculated according to Equation (1):

(1) $${\mathrm{SA}}_{\mathrm{DPPH}}=\frac{A_{\mathrm{blank}}-A_{\mathrm{sample}}}{A_{\mathrm{blank}}}\times 100$$

where *A_blank_* and *A_sample_* represent the absorbance of the DPPH solution alone and the DPPH–sample mixture, respectively. Three samples of each group were analyzed in triplicate to ensure reproducibility.

### 2.2. Cell Culture and Seeding on GGMA-Based Scaffolds

The human adipose-derived mesenchymal stem cells (hADMSCs) used in this work were obtained as previously reported [30]. hADMSCs cells were cultured in complete growth medium (MesenPRO RSTM medium, Thermo Fisher Scientific, Waltham, MA, USA, NYSE:TMO) complemented with 2.5 mM l-glutamine, 10% fetal bovine serum (FBS, Merck Life Science S.r.l., Milan, Italy), 1% penicillin/streptomycin/amphotericin (PSA, Merck Life Science S.r.l., Milan, Italy) and incubated overnight at 37 °C in a humidified atmosphere containing 5% CO_2_. Cells were grown to 70–80% confluence and the medium was replaced 2–3 times per week.

For seeding, 8 × 10^5^ hADMSCs resuspended in 100 µL of complete growth medium have been slowly plated on each scaffold, into 24 well plates, and incubated for 4 h at 37 °C in a humidified atmosphere with 5% CO_2_. Then, 500 µL of fresh complete growth medium were added to each well to completely cover the scaffold, and the plates were re-incubated, as above reported. The cells were grown on the scaffolds for 21 days by replacing the medium twice a week, and the biological analyses were performed at several timepoints (Day 1, Day 7, Day 14, and Day 21).

### 2.3. Cell Viability, Proliferation and Morphological Analysis of hADMSCs Cultured on GGMA-Based Scaffolds

Cell viability of hADMSCs cultured on various GGMA scaffold types was evaluated using the MTT assay [3-(4,5-dimethylthiazol-2-yl)-2,5-diphenyltetrazolium bromide] (Merck Life Science S.r.l., Milan, Italy) as previously described [31]. Briefly, after 1, 7, 14 and 21 days of culture, cell-seeded scaffolds were transferred to a new 24-well plate, 200 μL of MTT solution (1 mg/mL in FBS-free medium) was added and the plate incubated at 37 °C for 2 h. Afterwards, MTT solution was removed, formazan crystals were dissolved and the absorbance at 540 nm (OD540) was read using a microplate reader (AMR-100 Biosigma, Verona, Italy). Morphological analysis was performed by Hematoxylin and Eosin (H&E) staining. At each time point, scaffolds were rinsed in 1× PBS and fixed with 4% paraformaldehyde (PFA) for 30 min at room temperature. Samples were washed thrice with PBS, dehydrated in graded ethanol, cleared in xylene, and embedded in paraffin. Sections of 8 μm thickness were obtained using a semi-motorized rotary microtome (RM2245, Leica, Wetzlar, Germany), mounted on glass slides, and stored at room temperature. Subsequently, slides were dewaxed in xylene, rehydrated through graded ethanol (100% to 50%), and stained with hematoxylin (Merck Life Science S.r.l., Milan, Italy) for 5 min. After rinsing under running water, sections were stained with eosin (Merck Life Science S.r.l., Milan, Italy) for an additional 5 min, followed by dehydration (50% to 100% ethanol), clearing in xylene, and mounting with Biomount DPX (Biognost, Zagreb, Croatia). Microscopic evaluation was conducted using a NEXCOPE NE 900 microscope (TiesseLab, Milan, Italy), and images were captured digitally. For cell proliferation analysis, the number of hematoxylin-stained nuclei per section was quantified using Fiji ImageJ2 software (v2.9.0/1.54f). A minimum of five integral sections (*n* = 5) per scaffold type were analyzed. Each scaffold was assessed in biological triplicate.

### 2.4. Extracellular Matrix Mineralization of hADMSCs Cultured on GGMA-Based Scaffolds

Calcium deposits in cell-seeded GGMA scaffolds were assessed using Alizarin Red S (AR S) staining (Merck Life Science S.r.l., Milan, Italy), as previously described [16]. At each time point, scaffolds were fixed in 4% paraformaldehyde (PFA) for 30 min at room temperature, then washed thrice with 1X PBS. Samples were dehydrated through graded ethanol, cleared in xylene, and embedded in paraffin. Sections of 8 μm thickness were obtained using a microtome and mounted on glass slides. Slides were deparaffinized in xylene, rehydrated through graded ethanol, rinsed with distilled water for 5 min, and stained with 2% Alizarin Red S solution for 10 min at room temperature. Post-staining, sections were washed to remove excess dye, dehydrated in acetone and acetone-xylene (1:1), cleared in xylene, and mounted using Biomount DPX (Biognost, Zagreb, Croatia). Imaging was performed using a NEXCOPE NE 900 microscope, and digital images were captured for analysis. Quantification of calcium deposition was conducted using Fiji image J2 software (version 2.9.0/1.54f.) by measuring the mean gray value across at least five integral sections (*n* = 5) per scaffold type. Each scaffold was assessed in biological triplicate.

### 2.5. Gene Expression Analysis of hADMSCs Cultured on GGMA-Based Scaffolds

Quantitative real time-PCR (qRT-PCR) was performed as previously reported [32]. Concisely, total RNA was extracted from hADMSCs cultured on various GGMA-based scaffolds using the RNeasy Mini Kit (Qiagen, Milan, Italy). RNA concentration and purity were assessed via NanoDrop 1000 spectrophotometry (ThermoFisher, Waltham, MA, USA). For cDNA synthesis, 1 μg of total RNA per sample was reverse transcribed using the ImProm-II Reverse Transcription System (Promega, Milan, Italy).

qRT-PCR was performed on a QuantStudio 5 Real-Time PCR System (Applied Biosystems, Monza, Italy) using PowerUp™ SYBR™ Green Master Mix (Applied Biosystems, Monza, Italy). Primers targeting exon–exon junctions were designed using the Primer-BLAST (Primer3 version 2.5.0) tool to ensure specificity. Each reaction was run in triplicate, and gene expression was quantified using the 2^^−ΔΔCt^ method, with results expressed as fold change relative to control. Target genes included: Osterix (*SP7*), Collagen Type 1 (*COL1A1*), Alkaline Phosphatase (*ALPL*), Osteopontin (*SPP1*), Osteocalcin (*BGLAP*) and Osteonectin (*SPARC*). Glyceraldehyde-3-phosphate dehydrogenase (*GAPDH*) served as the endogenous reference gene. Primer sequences are detailed in Table 4.

### 2.6. Quantitative Assessment of Alkaline Phosphatase Activity

Alkaline phosphatase (ALP) activity was evaluated using a colorimetric assay performed on cell culture supernatants. hADMSCs (8 × 10^5^ cells) were seeded onto each scaffold and incubated for 1, 7, 14, and 21 days at 37 °C in a humidified atmosphere with 5% CO_2_. At each time point, conditioned media were collected for enzymatic analysis using a commercial alkaline phosphatase assay kit (ab83369, Abcam, Cambridge, UK), following the manufacturer’s instructions. Absorbance was measured at 405 nm with a microplate reader (AMR-100 Biosigma, Verona, Italy). All experiments were performed in biological triplicates, and for each biological replicate, five measurements were taken (*n* = 5) to ensure statistical robustness.

### 2.7. Statistical Analysis

For statistical analysis, data were analyzed either as raw data or as mean ± standard deviation (SD). Differences between several groups and timepoints of hADMSCs cultured on different GGMA scaffolds were analyzed by using one/two-way ANOVA with Tukey post hoc test. *p* < 0.05 was considered statistically significant. GraphPad Prism software version 9.0 (GraphPad Software Inc., San Diego, CA, USA) was used to generate graphs. All experiments were assessed in biological triplicate.

## 3. Results

### 3.1. 3D-Printed Scaffold Production and Characterization

**Figure 1:**
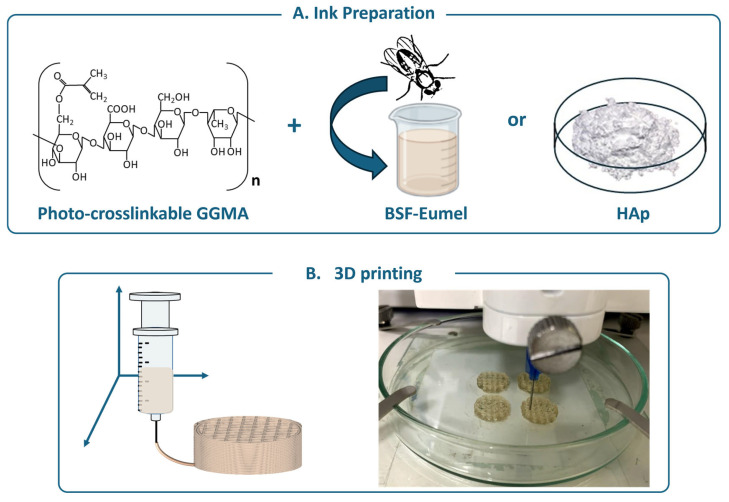
(**A**) Schematic representation of the main components constituting the ink: the polymer matrix [methacrylated gellan gum (GGMA)] functionalized with the Black Soldier Fly Eumelanin (BSF-Eumel) or hydroxyapatite (HAp). (**B**) 3D printing of GGMA4; GGMA4/BSF-Eumel and GGMA4/HAp30 scaffolds (**left**). Representative image of 3D-printed GGMA4/BSF-Eumel scaffolds (**right**).

Bioactive GGMA-based ink formulations incorporating either BSF-Eumel or HAp were developed and thoroughly characterized from physicochemical, morphological, and mechanical perspectives, as previously described [20]. A schematic overview of the process, from ink preparation to 3D printing, is presented in Figure 1.

These modified formulations facilitated the production of structurally coherent 3D-printed scaffolds, as validated through detailed morphological analysis [20]. Indeed, in this preliminary study, a printability optimization of GGMA inks was carried out to identify a formulation capable of maintaining high resolution and mechanical integrity after 3D printing. Two polymer concentrations, 2% and 4% *w*/*v*, were initially screened through a filament fusion test using a Rokit Invivo 4D2 bioprinter at controlled temperatures of 10 °C and 35 °C, respectively. The lower concentration (2% *w*/*v*) produced discontinuous, poorly defined filaments due to inadequate polymer chain entanglement and low yield stress, whereas the 4% *w*/*v* formulation (GGMA4) displayed continuous and self-supporting strands with excellent layer adhesion [20]. Jongprasitkul and co-workers provided a systematic rheological framework, demonstrating that the printability of GGMA is mainly governed by its shear-thinning behavior and the balance between ionic and photochemical crosslinking [33]. Rheologically, as evidenced by Jongprasitkul et al., GGMA at concentrations higher than 2% *w*/*v* was able to reach the critical entanglement regime, exhibiting pseudoplastic, shear-thinning flow behavior that enabled smooth extrusion under shear and rapid recovery after deposition [33]. However, concentrations above 4% *w*/*v* appeared to be excessively viscous and brittle, compromising print fidelity. By comparing the two papers, the mechanical and printability outcomes of D’Amora et al. [20] were consistent with Jongprasitkul’s rheological results: higher polymer and filler content may increase yield stress and elastic response, reinforcing layer fidelity but at the cost of higher extrusion resistance. Together, the two studies delineated a continuous optimization strategy, Jongprasitkul establishing the rheological printability window (≈2% *w*/*v* GGMA, moderate Ca^2+^, strong shear-thinning), and D’Amora demonstrating how bioactive reinforcement at 4% *w*/*v* GGMA (without pre-crosslinking) maintained print fidelity while improving stability and stiffness for BTE scaffolds [20,33].

The incorporation of bioactive fillers, HAp (10–30% *w*/*w*) and BSF-Eumel (0.3 mg/mL), further enhanced filament definition by slightly increasing viscoelasticity without impairing flow. Optimal printing conditions were obtained with 4% *w*/*v* GGMA inks printed at 35 °C and 35–45% infill, followed by dual crosslinking via UV exposure and mild CaCl_2_ treatment. This combination ensured balanced viscosity, yield stress, and mechanical robustness suitable for reliable 3D bioprinting. Among the tested conditions, scaffolds printed with the highest infill percentage (45%), referred to as GGMA4, demonstrated superior structural and physical performance for both bioactive additives.

A summary of the mechanical and physicochemical, including swelling behavior, stability profile and antioxidant properties, is reported in Table 5.

Dynamic mechanical analysis revealed that BSF-Eumel incorporation (GGMA4/BSF-Eumel) did not significantly enhance the mechanical properties, in terms of storage modulus, of the GGMA-based scaffolds. In contrast, increasing the HAp content to 30% (*w_HAp_*/*w_GGMA_*) in GGMA4/HAp30 resulted in a substantial rise in storage modulus. All the scaffolds were able to swell, even if the incorporation of HAp reduced water uptake due to increased network density [20]. Furthermore, the three scaffolds exhibited good stability over 28 days, with limited weight loss (<10%), though BSF-Eumel embedding allowed to maintain the highest mass retention [20].

The antioxidant properties of GGMA-based scaffolds were analyzed by the DPPH test (Table 5). GGMA4/BSF-Eumel scaffolds showed value of SA_DPPH_ higher than GGMA4 and GGMA4/HAp30 highlighting their potential to modulate reactive oxygen (ROS) species in accordance with previous studies [34].

### 3.2. Osteoconductive Response of hADMSCs Cultured on the Different Types of GGMA Scaffolds

To assess the biocompatibility of the tested scaffolds (GGMA4, GGMA4/HAp30 and GGMA4/BSF-Eumel), hADMSCs were cultured on their surfaces for 21 days. Cell adhesion, viability and proliferation were quantitatively analyzed at Days 1, 7, 14 and 21.

**Figure 2:**
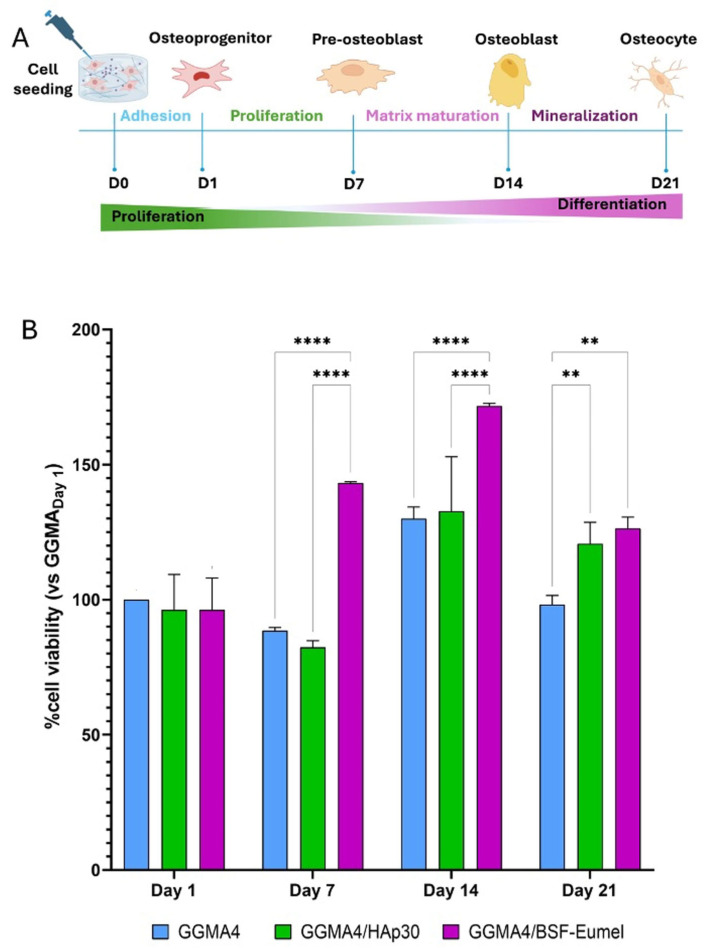
(**A**). Schematic representation of the seeding and culturing process of hADMSCs on the different scaffolds up to 21 days. (**B**). Cell viability analysis performed by MTT assay on hADMSCs cultured on GGMA4, GGMA4/HAp30, and GGMA4/BSF-Eumel scaffolds for 1, 7, 14, and 21 days. Data are reported as the mean percentage ± standard deviation obtained from five different samples (*n* = 5) of each type compared to the control group (GGMA4) on day 1. Statistical significance was determined by one-way ANOVA with Tukey’s post hoc test. The symbols denote: ** *p* < 0.01 and **** *p* < 0.0001.

The MTT assay results confirmed that all scaffolds tested, GGMA4, GGMA4/HAp30, and GGMA4/BSF-Eumel, were cytocompatible and non-cytotoxic, with comparable cell viability observed across all different biomaterials at Day 1. From Day 7 onward, however, some differences emerged: GGMA4/BSF-Eumel exhibited the most significant increase in cell viability, compared to both GGMA4 and GGMA4/HAp30, particularly by Day 21. GGMA4/HAp30 also enhanced cell viability compared to neat GGMA4 (control), although its effect was less significant than that of the BSF-Eumel scaffold (Figure 2).

**Figure 3:**
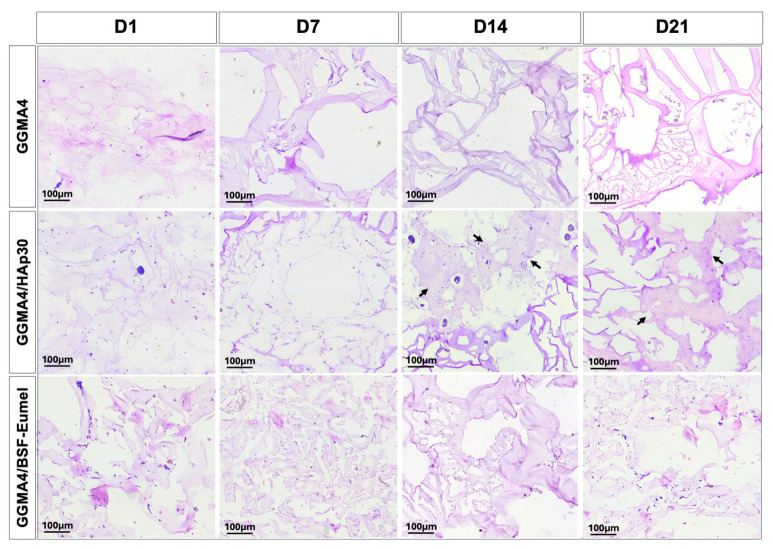
Representative images of H&E staining performed on hADMSCs cultured on GGMA4, GGMA4/HAp30, and GGMA4/BSF-Eumel for 1, 7, 14 and 21 days. Black arrows indicate areas of bone-like matrix deposition. Scale bars: 100 µm.

To evaluate the morphological behavior of hADMSCs within different GGMA-based scaffolds, a histological analysis using H&E staining was performed (Figure 3).

The results confirmed that hADMSCs adhered to and infiltrated all scaffold types (GGMA4, GGMA4/HAp30, and GGMA4/BSF-Eumel). However, cell density was notably greater in GGMA4/HAp30 and GGMA4/BSF-Eumel across all time points compared to the neat GGMA scaffold, suggesting enhanced cellular recruitment and retention induced by scaffold functionalization. At Days 14 and 21, GGMA4/HAp30 exhibited histological evidence of bone-like matrix deposition, indicated by the presence of organized areas and structural signs characteristic of osteogenic progression (black arrows, Figure 3). This feature was not observed in either GGMA4 or GGMA4/BSF-Eumel scaffolds, suggesting that HAp plays a critical role in promoting early mineralization and bone matrix formation.

**Figure 4:**
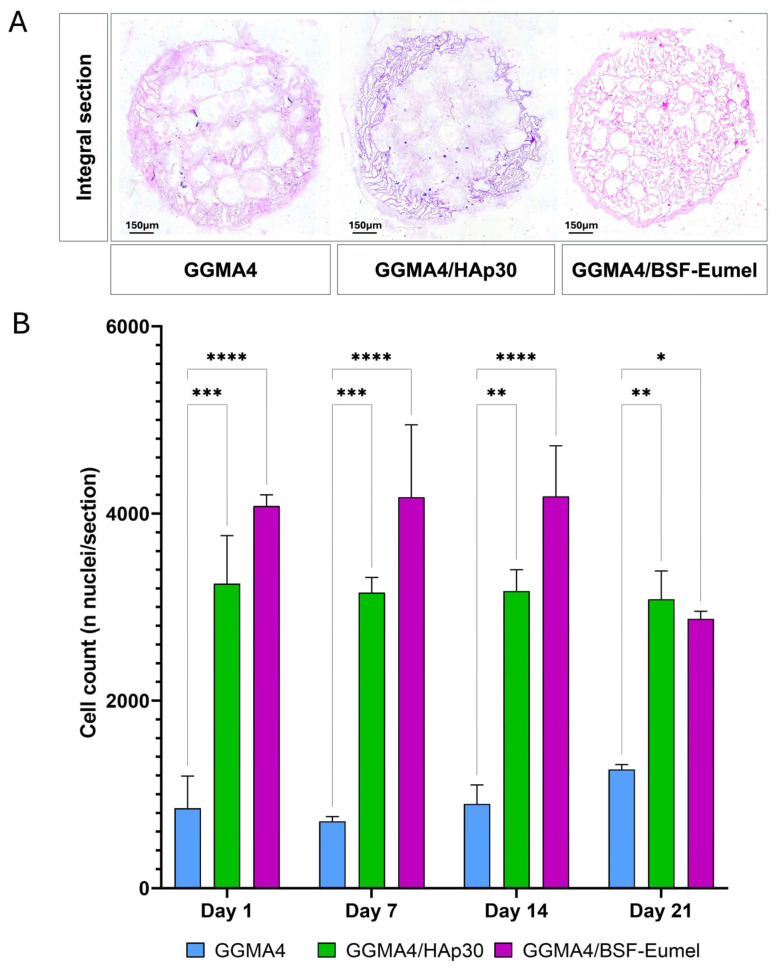
(**A**). Representative images of H&E staining of an integral section (diameter (⌀) = 13 mm and thickness (t) = 3 mm) of the different types of GGMA scaffold. Scale bar: 150 µm. (**B**). Graphical representation of nuclei counting performed on at least 5 different integral sections (*n* = 5), for each different type of scaffold, at each time point. Three scaffolds for each type were counted (biological triplicate). Statistical significance was determined by one-way ANOVA with Tukey’s post hoc test. The symbols denote: * *p* < 0.05, ** *p* < 0.01, *** *p* < 0.001, and **** *p* < 0.0001.

To further assess scaffold-induced cell proliferation, hADMSCs cultured within the GGMA4, GGMA4/HAp30, and GGMA4/BSF-Eumel scaffolds were evaluated using nuclei quantification in H&E-stained histological sections (Figure 4).

The results revealed that both GGMA4/HAp30 and GGMA4/BSF-Eumel scaffolds supported a sustained increase in cell number from Day 1 through Day 21, indicating their capacity to promote hADMSCs proliferation over time. GGMA4/HAp30 exhibited the most consistent gradual increase across all time points, while GGMA4/BSF-Eumel also showed a significant increase in cell density during the early and mid-stages of culture. However, a slight, non-significant reduction in the number of visible nuclei was observed in GGMA4/BSF-Eumel at Day 21 compared to Day 14, suggesting a potential plateau in proliferative activity or the start of differentiation-related processes. In contrast, the GGMA4 control scaffold maintained a consistently lower cell count throughout the culture period, highlighting the advantageous effect of scaffold functionalization on cellular behavior.

### 3.3. Osteoinductive Response of hADMSCs Seeded on the Different Types of GGMA-Based Scaffolds

**Figure 5:**
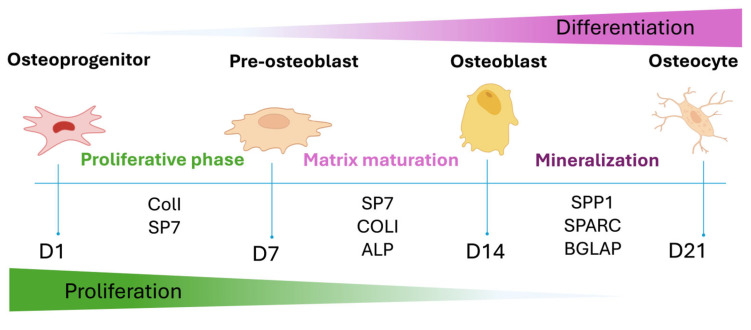
Graphical representation of the osteogenic differentiation of hADMSCs and the most important osteogenic markers expressed during the different phases.

To assess the osteoinductive potential of GGMA-based scaffolds, ECM mineralization and osteogenic gene expression were investigated. Figure 5 depicts a schematic overview of the temporal progression of hADMSC osteogenic differentiation, highlighting the sequential expression of key genetic markers involved in ECM production, mineralization, and lineage commitment.

Expression of *COL1* and *SP7* marks the initial commitment toward the osteoblastic lineage (Early phase). These genes are associated with early ECM organization and transcriptional regulation of bone-specific genes. As differentiation progresses into the mid phase, *ALPL* expression rises in parallel with *SP7* activity, indicating ECM maturation and the early phase of matrix calcification. The late phase is characterized by the upregulation of *SPP1*, *SPARC*, and *BGLAP* all of which are associated with terminal differentiation, matrix remodeling and mineral deposition.

**Figure 6:**
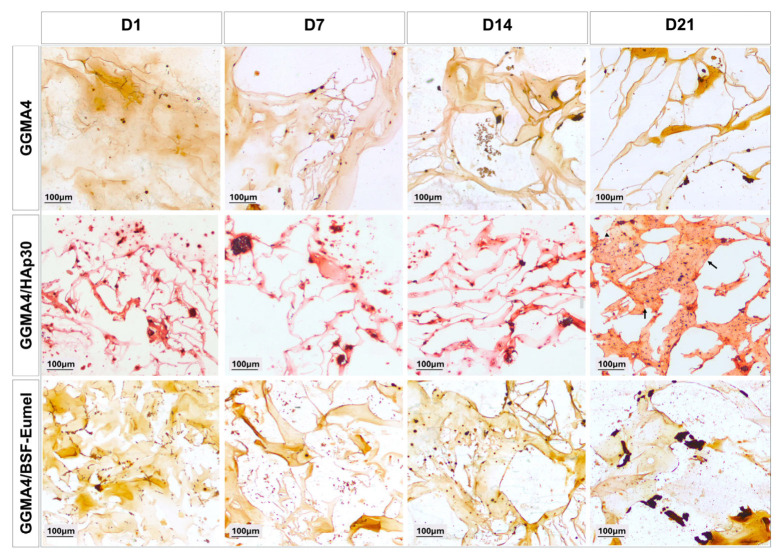
Representative images of AR S staining performed on hADMSCs cultured on GGMA4, GGMA4/HAp30, and GGMA4/BSF-Eumel for 1, 7, 14 and 21 days. Black arrows highlight regions resembling bone-like tissue. Scale bars: 100 µm.

Alizarin Red S (ARS) staining was used to assess calcium deposition and ECM calcification over time. As shown in Figure 6, all scaffold types revealed progressive mineralization from Day 1 to Day 21. Particularly, GGMA4/HAp30 and GGMA4/BSF-Eumel scaffolds exhibited significantly higher levels of mineral deposition compared to the neat GGMA4, as indicated by the presence of dense red-brown extracellular calcium aggregates visible in the Alizarin Red S-stained sections. By Day 21, GGMA4/HAp30 scaffolds showed histological features resembling bone-like tissue (indicated by black arrows), a response not visible in GGMA4 and GGMA4/BSF-Eumel scaffolds.

**Figure 7:**
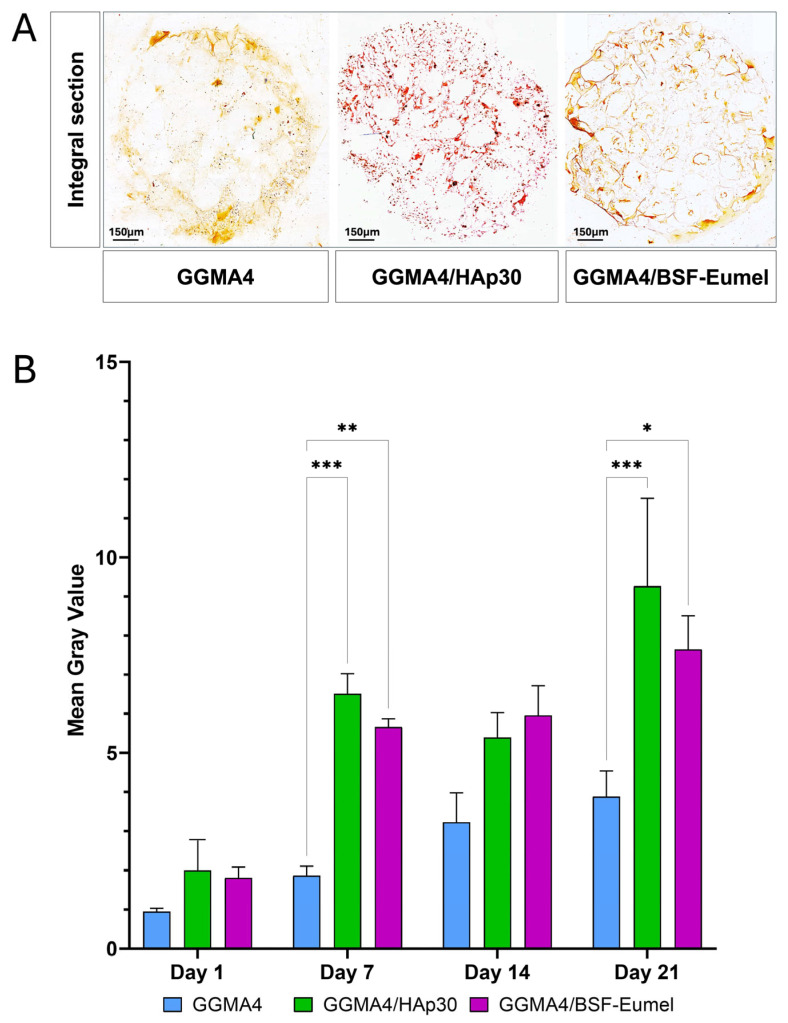
(**A**). Representative images of AR S staining of an integral section (diameter (⌀) = 13 mm and thickness (t) = 3 mm) of the different types of GGMA scaffold. Scale bar: 150 µm. (**B**). Quantitative evaluation of the AR S images by measuring the mean gray value of integral scaffold section. At least 5 different integral sections (*n* = 5), for each different type of scaffold, at each time point were quantified. Three scaffolds for each type were counted (biological triplicate. Statistical significance was determined by one-way ANOVA with Tukey’s post hoc test. The symbols denote: * *p* < 0.05, ** *p* < 0.01, and *** *p* < 0.001.

To perform a quantitative analysis of the matrix mineralization in all the GGMA scaffolds tested, the mean gray value of the acquired AR S images was measured. The results reported in Figure 7 confirmed an increase in mineralization over time, from day 1 to day 21, in all three scaffolds investigated, although this increase was statistically significant only in the GGMA4/HAp30 and GGMA4/BSF-Eumel scaffolds. These data also suggested that the mineralization level of GGMA4/HAp30 and GGMA4/BSF-Eumel scaffolds are very similar although in GGMA4/HAp30 scaffold is present the hydroxyapatite, the main mineral component of native bones.

**Figure 8:**
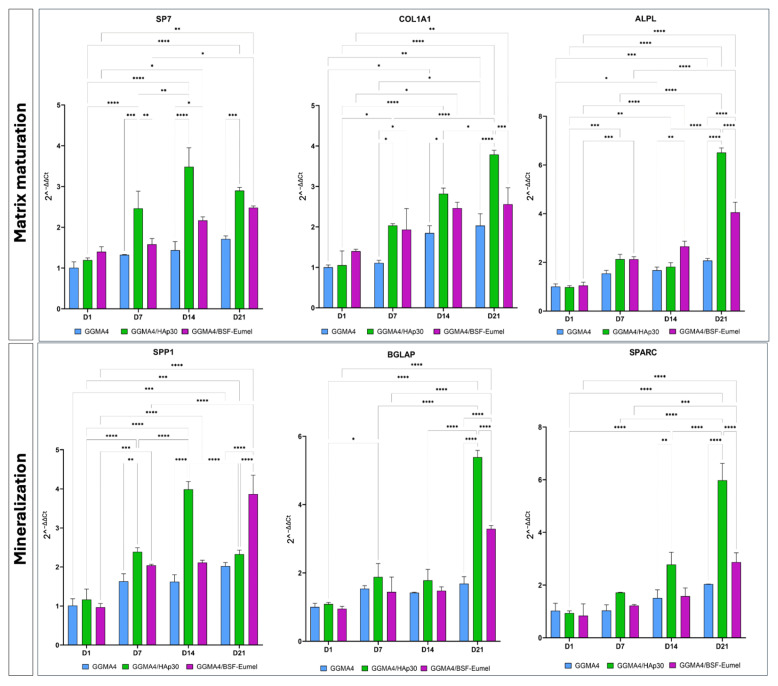
Gene expression profile of hADMSCs cultured on GGMA4, GGMA4/HAp30, and GGMA4/BSF-Eumel for 21 days performed with specific early and late markers of osteogenic differentiation: *SP7*, *COL1A1*, *ALPL*, *SPP1*, *BGLAP* and *SPARC*. GAPDH was used as an endogenous control. Statistical significance was determined by two-way ANOVA with Tukey’s post hoc test. The symbols denote: * *p* < 0.05, ** *p* < 0.01, *** *p* < 0.001, and **** *p* < 0.0001.

To further evaluate the osteogenic phenotype of hADMSCs after 21 days of culturing on the different types of scaffolds, qRT-PCR analysis with specific markers of matrix maturation (*SP7*, *COL1A1*, *ALPL*) and mineralization (*SPP1*, *BGLAP*, *SPARC*) phases was carried out (Figure 8).

The qRT-PCR data display that the expression levels of all matrix maturation and mineralization markers increase over time in GGMA4/HAp30 and GGMA4/BSF-Eumel compared to GGMA4, although the largest increase occurs between 14 and 21 days. Furthermore, it can be noted that, at 21 days, the levels of *COL1A1*, *ALPL*, *BGLAP* and *SPARC* in GGMA4/HAp30 are markedly higher than GGMA4/BSF-Eumel, probably due to the presence of hydroxyapatite which better mimics the ECM of native bone.

**Figure 9:**
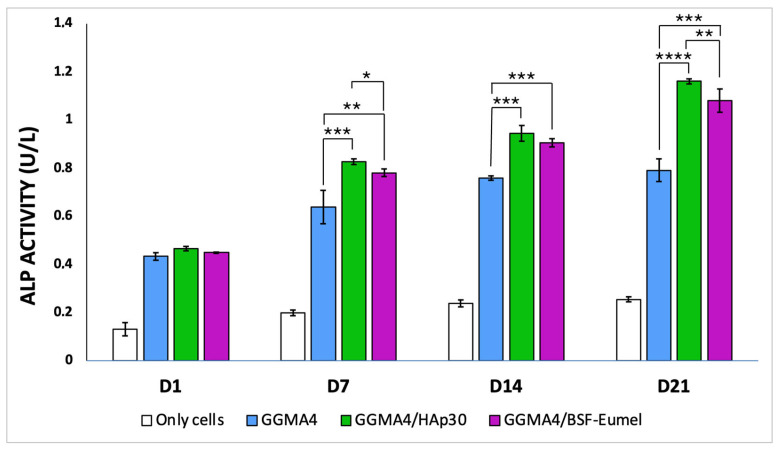
Bar graph illustrating ALP enzymatic activity (U/L) measured from culture supernatants of hADMSCs (Only cells) and hADMSCs seeded on GGMA4, GGMA4/HAp30, and GGMA4/BSF-Eumel scaffolds at Days 1, 7, 14, and 21. Data are represented as mean ± SD; all experiments were performed in biological triplicate, with five measurements taken for each replicate (*n* = 5). Statistical significance was determined by one-way ANOVA with Tukey’s post hoc test. The symbols denote: * *p* < 0.05, ** *p* < 0.01 and *** *p* < 0.001, and **** *p* < 0.0001.

In addition to analyzing *ALPL* mRNA transcript expression, the corresponding enzymatic activity was quantitatively measured over a 21-day period using an absorbance-based assay. This evaluation, conducted on the three different types of scaffolds (GGMA4, GGMA4/HAp30, and GGMA4/BSF-Eumel) was performed following the collection of culture supernatants. As shown in Figure 9, in all analyzed scaffolds there is a progressive increase in ALP levels over time, consistent with enhanced cellular activity and osteogenic differentiation.

Among the three, GGMA4/HAp30 demonstrates the highest ALP activity at each time point, suggesting it most effectively promotes osteoblast function and mineralization, likely due to the bioactivity of hydroxyapatite. GGMA4/BSF-Eumel exhibits intermediate activity, indicating that eumelanin incorporation might contribute to osteoinductive potential, possibly via antioxidant or biochemical signaling pathways.

## 4. Discussion

This study highlights the distinct and complementary roles of BSF-derived eumelanin and hydroxyapatite in enhancing the performance of GGMA-based inks for BTE. Each additive contributes uniquely to the scaffold’s mechanical, biochemical, and biological properties, shaping its overall functionality and therapeutic potential.

The mechanical reinforcement observed in GGMA4/HAp30 scaffolds aligns with the established role of mineral fillers in stiffening polymer matrices. The significant increase in storage modulus confirms that HAp integration improves scaffold rigidity, a critical factor in guiding stem cell fate and promoting tissue regeneration, particularly in mechanically sensitive environments [20]. In contrast, BSF-Eumel integration did not enhance mechanical strength, suggesting its primary contribution lies in biochemical modulation rather than structural reinforcement.

Notably, GGMA4/BSF-Eumel scaffolds exhibited superior long-term stability in culture medium. This enhanced durability is likely due to the eumelanin’s rich chemical structure, especially its carboxyl and catechol groups, which can chelate metal ions and stabilize the negatively charged polymer network. These interactions may reduce scaffold degradation and support sustained performance in both in vitro and in vivo environments [24]. Meanwhile, the reduced swelling observed in HAp-containing scaffolds suggests that inorganic fillers restrict polymer chain mobility, improving dimensional stability but potentially limiting nutrient diffusion and cell infiltration, factors that merit further investigation.

Biocompatibility assessments revealed that all scaffold variants were non-cytotoxic and supported hADMSCs adhesion and viability [35,36]. However, differences in cellular behavior emerged over time. GGMA4/BSF-Eumel scaffolds promoted early and robust cell proliferation, likely driven by eumelanin’s antioxidant and signaling properties [25]. GGMA4/HAp30 scaffolds, while slower to initiate proliferation, demonstrated consistent growth and histological evidence of bone-like matrix deposition by Day 21, underscoring HAp’s osteoconductive potential.

Nuclei quantification further supported these trends. GGMA4/BSF-Eumel showed strong early proliferation, followed by a slight decline at Day 21, possibly indicating a shift toward differentiation. GGMA4/HAp30 maintained a steady upward trajectory, suggesting sustained proliferative support and scaffold maturation. These observations point to a complementary biofunctional role: BSF-Eumel enhances early metabolic activity and cell viability, while HAp supports long-term proliferation and osteogenic differentiation [20,37,38,39].

The osteoinductive potential of both scaffold types was confirmed through ARS staining, qRT-PCR, and ALP enzymatic activity. GGMA4/HAp30 scaffolds exhibited dense calcium deposition and bone-like histological features, consistent with HAp’s role in promoting matrix mineralization and transcriptional regulation of osteogenic genes.

These findings demonstrate the distinct and complementary roles of HAp and BSF-Eumel in enhancing GGMA-based scaffolds for BTE. Each additive contributes uniquely to the scaffold’s mechanical, biochemical, and biological properties, shaping its overall functionality.

HAp, a well-established osteoconductive and osteoinductive material, mimics the mineral phase of bone and releases calcium and phosphate ions that activate key signaling pathways involved in osteogenesis. Its inclusion in polymer scaffolds consistently improves mineralization and mechanical strength, as confirmed by the dense calcium deposition and bone-like histological features observed in GGMA4/HAp30 scaffolds. These effects are further supported by literature demonstrating HAp’s efficacy in enhancing both mechanical and biological properties of polymer-based scaffolds and its synergistic potential when combined with bioactive molecules [40,41,42].

In contrast, BSF-Eumel contributes to osteogenic differentiation through a distinct, multifunctional mechanism that extends beyond mineral induction. Unlike HAp, which provides a static source of mineral ions, BSF-Eumel acts as an active biointerface within the hydrogel matrix, orchestrating multiple biochemical and mechanotransductive pathways.

Chemically, BSF-Eumel is a heterogeneous biomaterial enriched with catechol and quinone groups, conferring redox activity, metal-chelating capacity, and surface charge polarity. These properties allow BSF-Eumel to dynamically modulate the cellular microenvironment, influencing both mechanical and biochemical signaling pathways critical for osteogenic commitment. At the cell–material interface, the negatively charged and aromatic-rich surface of BSF-Eumel likely enhances the adsorption of adhesive proteins such as fibronectin and vitronectin from the serum. This enriched protein layer facilitates integrin clustering and activation of focal adhesion kinase (FAK), initiating downstream MAPK/ERK signaling cascades, mechanotransductive events known to regulate osteogenic gene expression, including RUNX2, COL1A1, and ALP [43].

Beyond mechanical signaling, BSF-Eumel’s ion-binding capacity plays a pivotal role in chemical modulation. Its ability to chelate Ca^2+^ imay create localized calcium-rich microdomains that stimulate BMP2 expression and Smad phosphorylation, key events in the BMP/Smad pathway that promote osteogenic differentiation and matrix mineralization [44,45]. Simultaneously, its antioxidant properties help maintain intracellular redox balance, mitigating oxidative stress that can otherwise inhibit BMP and Wnt/β-catenin signaling. Oxidative destabilization of β-catenin and suppression of Smad activation are known barriers to osteogenesis [46], and BSF-Eumel’s redox buffering may help preserve these pathways.

The interplay between integrin–FAK signaling and Wnt/β-catenin activity further underscores BSF-Eumel’s role in promoting osteogenesis. Mechanical signals generated at BSF-Eumel-rich interfaces enhance cytoskeletal tension, facilitating β-catenin nuclear translocation, a critical step in the transcription of osteogenic markers such as BGLAP and SPP1 [47]. Moreover, BSF-Eumel’s ability to chelate trace metal ions (e.g., Fe^3+^, Cu^2+^, Zn^2+^) may influence metalloprotein activity and enzymatic cofactors essential for matrix maturation and cell function.

Interestingly, despite GGMA4/HAp30 scaffolds showing stronger transcriptional and enzymatic markers of osteogenesis, Alizarin Red S quantification revealed comparable levels of mineral deposition between the two scaffold types. This suggests that BSF-Eumel may support mineralization through indirect mechanisms, such as enhancing cell viability and creating a favorable biochemical environment, rather than through direct ion release. The similar mineralization outcomes, despite differing gene expression profiles, highlight the complexity of scaffold–cell interactions and the need to consider both functional and structural contributions when interpreting osteogenic performance.

Importantly, BSF-Eumel is derived sustainably from black soldier fly by-products, offering both environmental and ethical advantages within a circular bioeconomy framework [25].

Unlike synthetic or microbial melanins, BSF-Eumel benefits from its integration with GGMA, which enhances mechanical stability and compatibility with scaffold fabrication techniques. This hybrid composition offers a unique advantage in terms of processability and structural integrity, distinguishing it from other melanin-based materials.

The comparative evaluation of GGMA4/HAp30 and GGMA4/BSF-Eumel scaffolds revealed a compelling divergence between cell viability and osteogenic functionality, which reflects the distinct biofunctional roles of each component. GGMA4/BSF-Eumel scaffolds exhibited the highest cell viability, likely due to the bioactive and antioxidant characteristics of eumelanin. Eumelanin has been shown to possess free radical scavenging and redox-modulating properties, which can mitigate oxidative stress and promote a favorable microenvironment for cell proliferation [27]. Additionally, GGMA contributed mechanical support and biocompatibility, further enhancing cell retention and survival as also previously showed [48].

In contrast, GGMA4/HAp30 scaffolds, despite slightly lower viability, appear to provide stronger osteoinductive cues. HAp is well documented for its ability to mimic the mineral phase of bone and release calcium ions, which are critical for osteogenic signaling pathways such as CaSR-JAK2/STAT3 and Wnt/β-catenin [49,50]. These cues facilitate cell commitment toward the osteoblastic lineage, as reflected in the upregulation of osteogenic genes and ALP activity. This contrast highlights the distinct biofunctional roles of each scaffold component and underscores the importance of evaluating both survival and lineage-specific outcomes in regenerative strategies. BSF-Eumel’s ability to scavenge ROS supports cell survival under stress conditions, while its immunomodulatory potential may regulate the early inflammatory phase of bone repair and promote scaffold integration. These features, combined with its biochemical signaling capabilities, suggest that BSF-Eumel contributes to a supportive microenvironment that enhances cell viability and early differentiation.

Taken together, these findings suggest that, while HAp drives osteogenic commitment through mineral-based signaling, BSF-Eumel contributes to a supportive microenvironment that enhances cell survival, modulates immune responses, and engages redox-sensitive signaling pathways. Rather than serving as a substitute for HAp, BSF-Eumel functions as a synergistic agent that broadens the functional scope of scaffold design.

## 5. Conclusions

In conclusion, scaffold functionalization with HAp and BSF-Eumel offers synergistic advantages, combining mechanical strength with bioactive modulation. These findings pave the way for the development of advanced composite biomaterials tailored for orthopedic and regenerative applications. Future studies could explore hybrid formulations that integrate both components to balance viability and differentiation, potentially optimizing outcomes for complex bone regeneration scenarios.

## Figures and Tables

**Table 1:** Nomenclature and chemical composition of the different ink formulations.

Ink	GGMA (% *w*/*v*)	BSF-Eumel (mg/mL)	HAp (% *w*/*w_GGMA_*)	Nomenclature
**GGMA**	4	-	-	GGMA4
**GGMA/BSF-Eumel**		0.3	-	GGMA4/BSF-Eumel
**GGMA/HAp**		-	30	GGMA4/HAp30

**Table 2:** Printing parameters for the realization of GGMA-based scaffolds.

Printing Parameters						
Scaffolds	Needle (mm)	Layer Thickness (mm)	Print Speed (mm/s)	Temperature (°C)	Pattern	Infill (%)
**GGMA**	0.41	0.3	3	35	Grid	45
**GGMA/BSF-Eumel**						
**GGMA/HAp30**						

**Table 3:** Parameters used for the post processing of the GGMA-based scaffolds.

Post Processing					
UV Crosslinking			CaCl_2_ Crosslinking		
Wavelength (nm)	Power Source (J/cm^2^)	Time (min)	CaCl_2_ (% *w*/*v*)	Time (min)	Temperature (°C)
365	10	10	0.05	10	25

**Table 4:** Primer sequences.

Gene	Forward	Reverse
***SP7***	TGCTTGAGGAGGAAGTTCACTATG	TGCCCAGAGTTGTTGAGTCC
***COL1A1***	CCGGAAACAGACAAGCAACCCAAA	AAAGGAGCAGAAAGGGCAGCATTG
***ALPL***	GACCCTTGACCCCCACAAT	CGCCTCGTACTGCATGTCCCCT
***SPP1***	AGTTTCGCAGACCTGACATCCAGT	TTCATAACTGTCCTTCCCACGGCT
***BGLAP***	GGCAGCGAGGTAGTGAAGAG	GATGTGGTCAGCCAACTCGT
***SPARC***	TTCTGCCTGGAGACAAGGTGCTAA	TCTGTTACTTCCCTTTGCCCACCT
***GAPDH***	TGTGAACGGATTTGGCCGTA	ACTGTGCCGTTGAATTTGCC

**Table 5:** Effect of BSF-Eumel and HAp on mechanical and physicochemical properties of the scaffolds.

Sample	Storage Modulus (E’, kPa) [20]	Swelling Behavior (Q, mg/mg) [20]	Stability (Relative Weight, W_R_%) [20]	DPPH Scavenging Activity (SA_DPPH_ %)
**GGMA4**	9.3 kPa	Highest ≈ 28.4 ± 2.9 (after 24 h), de-swelling after 7 days	Gradual ~10% weight loss over 28 days; moderate stability	9.0 ± 1.3
**GGMA4/HAp30**	32.5 kPa (≈3.5 × GGMA4)	Lowest ≈ 19.8 ± 1.3 after 24 h) due to reduced chain mobility	Slight initial burst loss then stable; total ~10% mass loss after 28 days	8.4 ± 0.6
**GGMA4/BSF-Eumel**	12.7 kPa (similar to GGMA4, no significant increase)	26.9 ± 0.6 (after 24 h); sustained swelling up to 28 days	Highest stability: minimal weight loss due to negatively charged eumelanin groups enabling ionic stabilization	27.5 ± 5.4

## Data Availability

The data that support the findings of this study are available from the corresponding author upon reasonable request.

## Abbreviations

The following abbreviations are used in this manuscript:

BSF-Eumel	Black Soldier Fly Eumelanin
hADMSCs	Human adipose-derived mesenchymal stem cells
GG	Gellan gum
HAp	Hydroxyapatite
BSF-eumel	Eumelanin derived from black soldier fly
MSCs	Mesenchymal stem cells
BTE	Bone tissue engineering
ECM	Extracellular matrix
3D	Three-dimensional
CAD	Computer-aided design
H&E	Hematoxylin and Eosin
AR S	Alizarin Red S
PFA	Paraformaldehyde
qRT-PCR	Quantitative real time-PCR
SP7	Osterix
Col1A1	Collagen Type 1
ALPL	Alkaline Phosphatase
SPP1	Osteopontin
BGLAP	Osteocalcin
SPARC	Osteonectin
GAPDH	Glyceraldehyde-3-phosphate dehydrogenase
DMA	Dynamic mechanical analysis
